# Secular trends in osteoporosis prevalence at first presentation over 26 years in a regional Irish DEXA registry: a retrospective repeated cross-sectional analysis

**DOI:** 10.1007/s11657-026-01764-z

**Published:** 2026-08-19

**Authors:** Azrin Muslim, Eleanor Fallon, Nur Atikah Mohd Asri, Aine Costelloe, Tina Sheehy, Mary Griffin, Declan Lyons

**Affiliations:** 1 Research Directorate (HSE Mid-West), St Camillus Hospital, Shelbourne Road, Limerick, Ireland; 2 Clinical Age Assessment Unit, University Hospital Limerick, Dooradoyle, Limerick, Ireland

**Keywords:** Osteoporosis, DEXA, Secular trends, Ireland, Bone mineral density

## Abstract

***Summary*:**

Declining osteoporosis prevalence at first DEXA presentation over 26 years in a regional Irish registry suggests improving baseline skeletal health independent of demographic shifts. If sustained, these trends may temper projected fracture burden, with implications for prevention strategies and health-system planning.

**Purpose:**

Despite decades of investment in fracture prevention and growing clinical awareness of osteoporosis, whether the skeletal health of individuals presenting for assessment has actually improved remains unknown. We investigated secular trends in osteoporosis prevalence at first presentation using a regional Irish DEXA registry spanning 26 years.

**Methods:**

In a retrospective repeated cross-sectional analysis, we examined all DEXA scans performed between January 1998 and December 2024 at two hospitals serving the Midwest region of Ireland, restricting analyses to each individual’s first scan to minimise treatment and surveillance bias. Osteoporosis was defined as a T-score of − 2.5 or lower at the total hip or lumbar spine. Logistic regression assessed calendar year as the exposure and osteoporosis as the outcome, adjusted for age and sex. Direct age standardisation and 3-year moving averages were used to stabilise annual estimates.

**Results:**

Among 66,681 individuals undergoing a first DEXA scan (80.7% female), osteoporosis prevalence declined substantially. Hip osteoporosis fell from 19 to 7% and lumbar spine osteoporosis from 30 to 8% between 1998 and 2024. Each calendar year was associated with a 4.0% reduction in odds of hip osteoporosis (OR 0.960, 95% CI 0.957–0.964) and a 4.8% reduction in lumbar spine osteoporosis (OR 0.952, 0.949–0.955). These findings persisted after age standardisation and adjustment for scan volume.

**Conclusion:**

Osteoporosis prevalence at first presentation declined by approximately two-thirds over nearly three decades, pointing to a meaningful improvement in skeletal health at the point of clinical assessment, with potential implications for future fracture burden projections.

**Supplementary Information:**

The online version contains supplementary material available at 10.1007/s11657-026-01764-z.

## Introduction

Fragility fractures of the hip and spine remain among the most consequential complications of ageing, driving substantial morbidity, mortality, and healthcare expenditure worldwide [1]. Over the past three decades, European health systems have pursued coordinated strategies to address this burden, from nutritional policy to widening clinical awareness of osteoporosis and fracture risk [2, 3]. Yet whether these efforts have translated into measurable improvements in skeletal health at the point of first clinical assessment remains an open question.

Most large-scale epidemiological studies of osteoporosis have focused on fracture incidence or the management of treated populations [3–5]. These designs cannot separate genuine changes in baseline skeletal health from the effects of treatment, evolving referral behaviour, or improved survival. To detect true secular change, one must examine osteoporosis prevalence at the point of first clinical presentation: before treatment has begun.

University Hospital Limerick and St Camillus Hospital in the Midwest region of Ireland have maintained a comprehensive regional DEXA registry since the late 1990 s, offering an unusual vantage point: a single, stable referral system through which long-term trends in baseline skeletal status can be tracked at initial clinical assessment rather than estimated from population surveys. We used this resource to ask two questions: (1) has osteoporosis prevalence at first presentation changed over time, independent of age and sex and (2) do any observed changes persist after age standardisation and adjustment for scan volume?

## Methods

### Data source and study population

We analysed all DEXA scans performed at University Hospital Limerick and St Camillus Hospital from January 1998 to December 2024. Scans from 2025 were excluded because of partial-year incompleteness (*n* = 15), yielding a final analytic cohort of 66,681 individuals. These facilities serve the Midwest region of Ireland (population approximately 400,000), providing DEXA services for clinical referrals from primary care, hospital specialists, and fracture liaison services. The registry includes scan dates, demographic, anthropometrics, and T-scores at the hip and lumbar spine.

DEXA scans were performed using successive generations of densitometry systems over the study period, beginning with a Siemens/Lunar pencil-beam scanner in the late 1990s, followed by GE Lunar Prodigy and subsequently GE iDXA fan-beam systems. The young-adult reference database used for T-score calculation was the NHANES III reference population [6]. Throughout the study period (1998–2024), T-scores were calculated using the manufacturer’s implementation of the NHANES III reference dataset within the installed software versions, and no documented systematic changes to the reference database were identified.

Quality assurance procedures were conducted in accordance with manufacturer guidelines. Daily QA calibration was performed prior to patient scanning using a manufacturer-supplied phantom block containing bone-equivalent chambers of known mineral content. Calibration measurements were required to fall within ± 0.030 g/cm^2^ of expected values with linear correlation coefficients ≥ 0.99. Manufacturer-reported precision error for AP spine and femoral BMD measurements is approximately 1% coefficient of variation (0.010 g/cm^2^). QA results were trended longitudinally within the system software. When densitometry systems were replaced or upgraded, cross-calibration and routine phantom-based quality assurance procedures were undertaken according to manufacturer and service protocols to ensure continuity of BMD measurements across platforms.

Sex was recorded from DEXA scan data, missingness after restricting to first scans and excluding partial 2025 data was ~ 7.9% (*n* = 5299) which were treated as missing in complete-case analyses. The three sex categories in Table 1 sum to the total cohort *N* = 66,681 (see Table 1 footnote). Hip T-score (1.9%), lumbar spine T-score (0.2%), height (1.5%), and weight (1.6%) had missing values that were not imputed; records with missing hip or lumbar spine T-score were excluded via complete-case analysis in the regression models (analytic *N* = 60,339 for hip and 61,267 for lumbar spine models). Age at scan is deterministically derived from date of birth and scan date and had no missing values in the final analytic dataset.

**Table 1:** Baseline characteristics of individuals undergoing their first DEXA scan (1998–2024). Data shown as mean (SD) or *n* (%). Overall cohort *N* = 66,681 first-ever scans. Percentages for sex are of the total cohort (missing/other sex data ≈7.9%). Unknown values indicate missing data for that characteristic

	Overall (*N* = 66,681)
Characteristic	***N***= 66,681^a^
**Age at scan**	61.4 (13.2)
**Sex**	
Female	53,837 (80.7%)
Male	7545 (11.3%)
Unknown	5299
**Height**	163.5 (30.3)
Unknown	1026
**Weight**	70.6 (50.5)
Unknown	1035
**T-score hip**	−0.9 (1.3)
Unknown	1283
**T-score lumbar spine**	−0.8 (1.7)
Unknown	143

^a^Female, male, and unknown sex categories sum to the total cohort *N* = 66,681

### Outcomes

Osteoporosis was defined according to World Health Organization criteria as a T-score of − 2.5 or lower at the total hip or lumbar spine [7]. Separate binary indicators were created for hip osteoporosis and lumbar spine osteoporosis. Annual prevalence and mean T-scores were calculated for each skeletal site. T-scores were cleaned to remove formatting inconsistencies prior to numeric conversion.

### Covariates

Age at scan and sex were included as covariates. Calendar year of scan was treated as a continuous exposure variable.

### Statistical analysis

Annual mean T-scores and crude osteoporosis prevalence were calculated separately for hip and lumbar spine. Separate logistic regression models were fitted for hip and lumbar spine osteoporosis as binary outcomes, with calendar year as the primary exposure, adjusted for age and sex.

Direct age standardisation was performed using five age bands [8] (40–49, 50–59, 60–69, 70–79, and ≥ 80 years) with the pooled age distribution of the cohort as the reference, thereby focusing on individuals aged ≥ 40 years, as low bone mineral density in younger adults is typically attributable to secondary causes rather than reflecting secular trends in age-related skeletal health. In effect, this procedure estimates the osteoporosis prevalence that would have been observed in each calendar year had the age distribution remained constant across the study period.

Three-year moving averages were used to stabilise annual prevalence estimates for visualisation and did not influence model-based inference. To assess whether increasing scan volume and potential referral expansion influenced observed trends, sensitivity analyses were performed with additional adjustment for annual first-scan volume. All analyses were restricted to first recorded DEXA scans.

To contextualise these findings for health-system planning, exploratory scenario modelling was performed, translating the observed annual reduction in hip osteoporosis odds into illustrative projections of future hip fracture burden relative to a demographic-only trajectory based on population ageing, under three assumed translation fractions (25%, 50%, and 75%) linking the observed reduction in osteoporosis prevalence to a corresponding reduction in fracture risk; these projections are exploratory and non-causal (Supplementary Table S3). Analyses were performed using R version 4.5.1.

Missing data were present for sex (7.9%), hip T-score (1.9%), lumbar spine T-score (0.2%); age at scan had no missing values in the final analytic dataset. Hip T-score missingness increased modestly with age (0.5% in 40–49 years to 6.4% in ≥ 80 years), consistent with occasional clinical omission of hip measurements in frail older individuals. Missing sex was more common in the oldest age group (24.3% in ≥ 80 years). There was no evidence of substantial differential missingness by calendar year (Supplementary Table S1).

Records with missing hip or lumbar spine T-score, age at scan, sex, or annual scan volume were excluded via complete-case analysis (analytic *N* = 60,339 for hip and 61,267 for lumbar spine). In a sensitivity analysis, coding records with missing T-score as non-osteoporotic rather than excluding them produced identical odds ratios to three decimal places, confirming that this choice did not materially affect the estimated trends.

## Results

After excluding repeat scans and partial/incomplete data from 2025, the final analytic cohort comprised 66,681 individuals undergoing their first DEXA scan (Table 1).

Between 1998 and 2024, 66,681 individuals underwent a first DEXA scan (53,837 [80.7%] female, 7545 [11.3%] male, and 5299 [7.9%] with missing sex data). Hip T-scores were available for 65,398 individuals and lumbar spine T-scores for 66,538 individuals. Emphasis was placed on complete-year estimates, and three-year moving averages were used to stabilise annual trends. Baseline characteristics of the first-scan population are shown in Table 1.

**Fig. 1:**
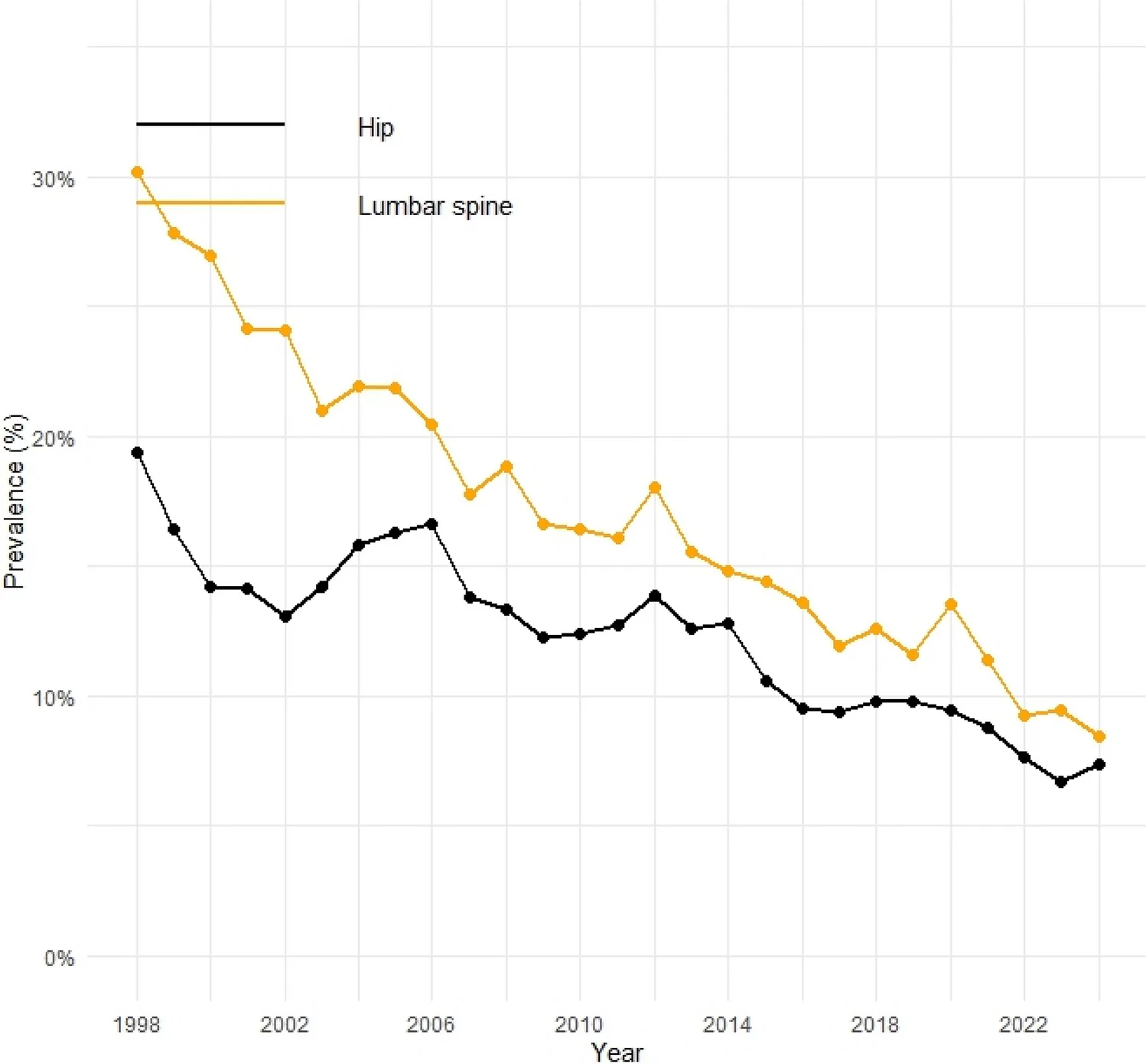
Crude osteoporosis prevalence at first DEXA scan by site (hip and lumbar spine), 1998–2024. Unadjusted prevalence (T-score ≤  − 2.5), full cohort

Crude prevalence of hip osteoporosis declined from 19.4% in 1998 to 7.4% in 2024, while lumbar spine osteoporosis declined from 30.2% to 8.4% over the same period (Fig. 1).

Mean T-scores at both skeletal sites increased progressively across calendar years (Supplementary Fig. S1).

In age- and sex-adjusted logistic regression models, calendar year was associated with lower odds of osteoporosis. The odds that an individual had osteoporosis at the hip decreased by 4.0% per year (OR 0.960, 95% CI 0.957–0.964). The odds that an individual had osteoporosis at the lumbar spine decreased by 4.8% per year (OR 0.952, 95% CI 0.949–0.955) (Table 2).

**Table 2:** Odds ratios (OR) and 95% confidence intervals (CI) per year for osteoporosis at first DEXA scan (hip and lumbar spine), 1998–2024. Models use complete cases with non-missing T-score, age at scan, sex, and annual scan volume (*N* = 60,339 for hip and 61,267 for lumbar spine). The overall cohort includes 66,681 first scans. All *p*-values were < 0.001

Site	Model	OR per year	95% CI	*p*-value
Hip	Crude	0.964	(0.960–0.967)	< 0.001
Hip	Age- and sex-adjusted	0.960	(0.957–0.964)	< 0.001
Hip	Age-, sex-, and volume-adjusted	0.969	(0.962–0.975)	< 0.001
Lumbar spine	Crude	0.952	(0.949–0.954)	< 0.001
Lumbar spine	Age- and sex-adjusted	0.952	(0.949–0.955)	< 0.001
Lumbar spine	Age-, sex-, and volume-adjusted	0.962	(0.957–0.968)	< 0.001

Logistic regression models were fitted using complete-case analysis, restricted to individuals with non-missing values for osteoporosis status (T-score ≤  − 2.5 at hip or lumbar spine), age at scan, sex, and annual first-scan volume. This resulted in analytic sample sizes of 60,339 for hip models and 61,267 for lumbar spine models, out of the overall cohort of 66,681 first scans.

**Fig. 2:**
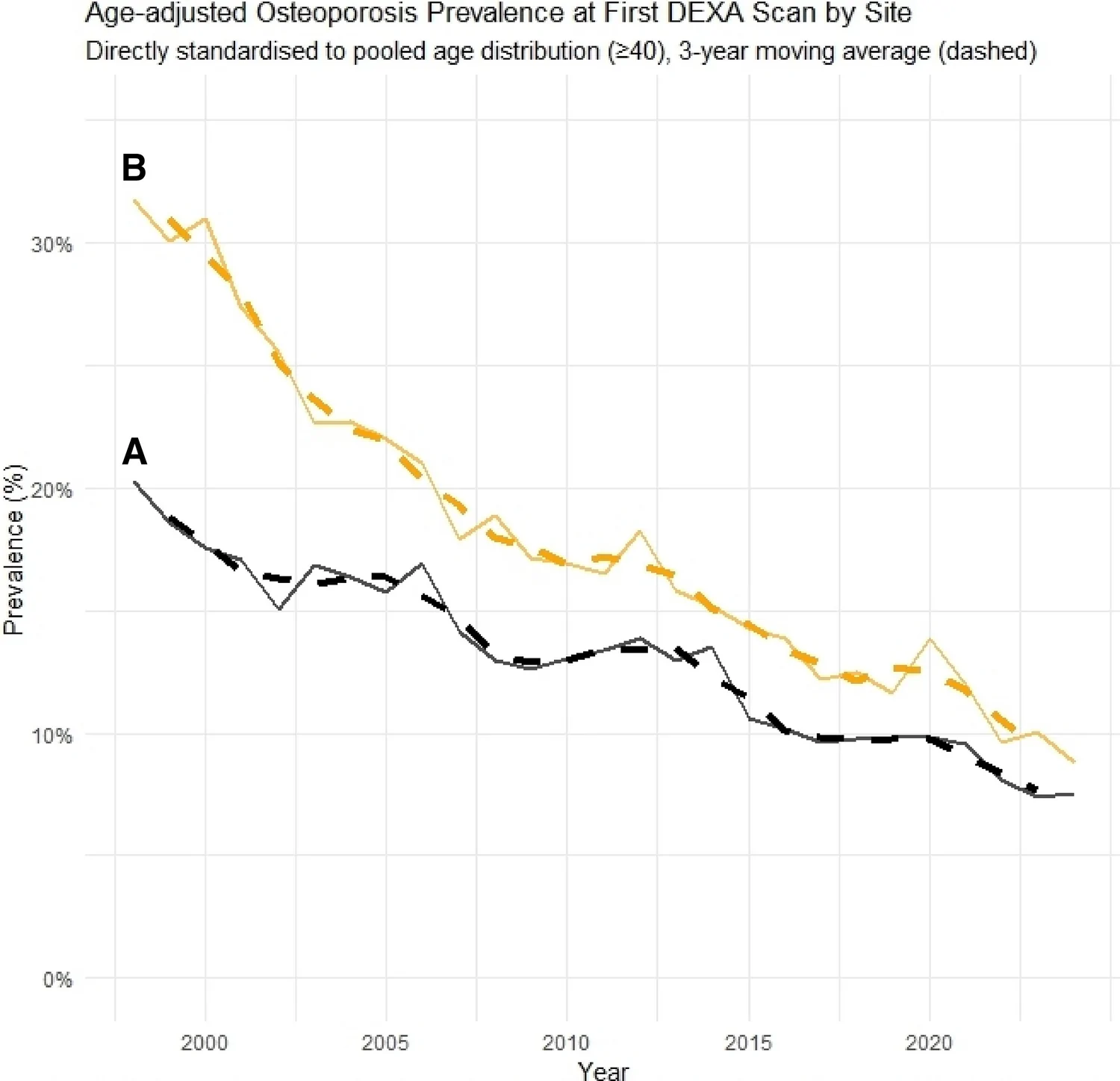
Age-standardised prevalence of hip (**A**) and lumbar spine (**B**) osteoporosis at first DEXA scan, 1998–2024. Direct standardisation to pooled age distribution; 3-year moving averages (dashed lines) may show minor boundary effects in the most recent years due to smoothing

After additional adjustment for annual first-scan volume, calendar year remained strongly associated with lower odds of osteoporosis (hip: OR 0.969 per year, 95% CI 0.962–0.975; spine: OR 0.962 per year, 95% CI 0.957–0.968). Independently of this model-based adjustment, directly age-standardised prevalence curves demonstrated parallel declines across the study period (Fig. 2).

After age standardisation, hip osteoporosis prevalence declined from approximately 19% in 1998 to 7% in 2022–2024, and lumbar spine osteoporosis from 30% to approximately 8%.

To examine whether temporal changes in bone mineral density were independent of shifts in age distribution, we calculated age-standardised mean T-scores at the hip and lumbar spine using direct standardisation with the pooled age-band distribution of the cohort (40–49, 50–59, 60–69, 70–79, and ≥ 80 years). Age-standardised mean T-scores increased steadily at both skeletal sites (Supplementary Figure S1). At the hip, standardised means improved from − 1.26 in 1998 to − 0.68 in 2024 (absolute increase 0.58 units). Lumbar spine values improved from − 1.66 to − 0.31 (absolute increase 1.35 units). Three-year moving averages demonstrated consistent upward trends over the study period.

Referral characteristics remained relatively stable over the 26-year period. The number of first scans increased from 5686 in 1998–2002 to 22,045 in 2018–2024. Mean age at first scan increased modestly across the study period, from approximately 60.0 years in 1998–2002 to 61.5 years by 2018–2024, arguing against referral of younger individuals as an explanation for the observed decline (Supplementary Figure S2). The proportion of females (among those with non-missing sex) remained consistently high across periods (84.9–94.6%), declining gradually from 94.6% in 1998–2002 to 84.9% in 2018–2024. Mean age at first scan increased modestly from 60.0 (SD 13.1) years in 1998–2002 to 61.5 (SD 13.2) years in 2018–2024. Among the 63,824 individuals aged ≥ 40 years with non-missing age, 51,685 (81.0%) were female. The relative stability in age and sex distribution over time (Supplementary Table S2) suggests minimal shifts in referral patterns explaining the observed declines.

In age- and sex-adjusted sensitivity analyses excluding the earliest years (1998–2002), the annual decline remained materially unchanged (hip OR per year 0.959; spine OR per year 0.956), supporting robustness against early-period instability.

Exploratory scenario modelling translating these findings into potential future fracture-burden implications is presented in Supplementary Table S3.

## Discussion

Over 26 years, osteoporosis prevalence at first clinical presentation in this regional DEXA registry declined substantially. The scale of this reduction, approximately two-thirds at the lumbar spine and more than half at the hip, represents a striking shift in the baseline skeletal health of individuals referred for assessment.

Declining age-standardised hip fracture incidence reported across several high-income countries in recent decades [9, 10] raises the possibility that the present findings reflect broader secular improvements in skeletal health.

Because analyses were restricted to first scans and adjusted for age and scan volume, the observed trends are unlikely to be explained by treatment effects, repeat surveillance, or changes in referral intensity alone, although residual selection bias cannot be fully excluded. Age standardisation was restricted to individuals aged ≥ 40 years using five bands, given that low BMD before this age is usually secondary to identifiable causes rather than reflecting population-level trends in skeletal health.

DEXA referral in the region has followed a consistent case-finding framework aligned with international guidelines rather than population screening, emphasising age and established fracture risk factors [11]. There is no evidence of a systematic expansion of referral to lower-risk individuals that would plausibly account for the magnitude and consistency of the observed decline.

However, a further consideration is that access to DEXA scanning was likely more limited in the earliest years of the registry than in later years, when capacity expanded substantially. If referral during this early, lower-capacity period was skewed toward individuals with more severe disease or a higher underlying fracture risk, this could contribute to an apparent decline in osteoporosis prevalence over time that is independent of any true secular change, and this possibility cannot be fully excluded from the available data. At the same time, first-scan volume increased more than three-fold between 1998–2002 and 2018–2024 without any corresponding shift toward a younger or lower-risk referred population, since the age and sex distribution remained relatively stable across calendar periods (Supplementary Table S2), and the age- and sex-adjusted annual decline in osteoporosis odds remained materially unchanged in sensitivity analyses excluding the earliest years (1998–2002).

It is important to recognise that reports of rising global fracture burden largely reflect population ageing and growth rather than worsening age-specific skeletal risk. International analyses confirm that while absolute fracture numbers have risen, age-standardised rates in many high-income settings have stabilised or fallen [4, 12]. The present study addresses a complementary question: whether skeletal health at first clinical assessment has itself improved, even as the absolute population burden continues to grow.

T-scores derived over extended periods can be influenced by changes in densitometer hardware, software versions, cross-calibration protocols, and reference databases [13]. Although daily phantom calibration and longitudinal quality control are standard practice, residual measurement drift cannot be entirely excluded. Lumbar spine values may also be artefactually elevated by degenerative change and vascular calcification in older adults, potentially masking true osteoporosis [13]. The concordant decline at the hip, a site less susceptible to such artefacts, supports the overall direction of the finding.

Restricting to first scans reduces bias from treatment initiation and monitoring. Although medication histories were incomplete and prior treatment cannot be entirely excluded, the relatively captive regional referral population and the role of DEXA as the gateway to osteoporosis assessment suggest that the majority of first scans were performed in treatment-naïve individuals.

There are several limitations in this study. The cohort comprises individuals referred for clinical assessment rather than a screening population, limiting inference about community prevalence. Fracture outcomes were not examined, though linkage to fracture and hospitalisation datasets is underway. Non-linear temporal trends are possible and warrant further investigation.

The scenario-modelling estimates (Supplementary Table S3) are illustrative and non-causal, and should be interpreted cautiously pending formal linkage to fracture outcomes.

## Conclusion

In a regional DEXA registry spanning nearly three decades and more than 66,000 first scans, osteoporosis prevalence at first clinical presentation declined markedly at both the hip and lumbar spine. The consistency of this decline across skeletal sites and after age standardisation points to a genuine improvement in baseline bone mineral density at initial evaluation. Although the causal drivers cannot be identified from these data, the findings are consistent with long-term improvements in the determinants of skeletal health across the life course.

These findings carry implications for fracture prevention and health-system planning. If the observed improvements in bone mineral density at presentation translate into sustained reductions in fragility fracture incidence, future fracture burden may be lower than projections based on historical osteoporosis prevalence would suggest. Linking this registry to fracture and hospitalisation outcomes will be essential to determine whether these improvements translate into fewer downstream clinical events.

The conventional expectation has been that increasing longevity would bring a rising burden of age-associated diseases, fractures among them [14]. The present findings, however, raise the possibility that age-specific fracture risk may itself be declining, meaning that projections based solely on demographic ageing may overestimate future fracture burden if baseline skeletal health continues to improve. As survival from cardiovascular disease and cancer has improved across high-income countries, attention has naturally shifted toward musculoskeletal conditions, including osteoporosis and fragility fractures [14–16]. Yet the decline in osteoporosis documented here challenges this prevailing narrative and suggests that skeletal health, like cardiovascular and cancer risk, may be modifiable at the population level. If so, future research and healthcare planning may need to direct greater attention towards other determinants of late-life morbidity, including falls-related syndromes, cognitive decline, multimorbidity, and the growing relative burden of neurodegenerative disease.

## Supplementary Information

Below is the link to the electronic supplementary material.

ESM 1 DOCX (124 KB)

ESM 2 DOCX (66.8 KB)

ESM 3 DOCX (18.1 KB)

ESM 4 DOCX (16.2 KB)

ESM 5 DOCX (398 KB)

## Data Availability

Individual participant data will not be made publicly available because the dataset is derived from a regional clinical DEXA registry and contains patient-level health information subject to institutional governance and data protection regulations under GDPR and the Health Research Regulations 2018. Anonymised aggregated data supporting the findings of this study, together with the statistical analysis code, may be made available upon reasonable request to the corresponding author. Access will require review and approval by the Health Service Executive (HSE) and compliance with institutional data governance procedures. Data will be available beginning 6 months after publication and will be retained for 7 years following study completion in accordance with HSE National Policy for Consent in Health and Social Care Research (2023). Requests should include a methodologically sound proposal and appropriate ethical approval where required.
