# Supplementary material for: Secular trends in osteoporosis prevalence at first presentation over 26 years in a regional Irish DEXA registry: a retrospective repeated cross-sectional analysis
Source: Arch Osteoporos. 2026 Aug 19;21(1):122. doi: 10.1007/s11657-026-01764-z (PMC13490005; doi:10.1007/s11657-026-01764-z)
Supplement: Supplementary file 1 [file 11657_2026_1764_MOESM1_ESM.docx]

**Supplementary Figure S1**

**Age-standardised mean T-scores at first DEXA scan, 1998–2024**

*Secular trends in osteoporosis prevalence at first presentation over 26 years in a regional Irish DEXA registry*

**Figure**

*Figure S1. Age-standardised mean hip (blue) and lumbar spine (orange) T-scores at first DEXA scan, 1998–2024. Solid lines represent annual age-standardised means; dashed lines represent 3-year moving averages. Direct standardisation was performed using the pooled age-band distribution of the cohort (40–49, 50–59, 60–69, 70–79, and ≥80 years).* Hip T-scores improved from −1·26 in 1998 to −0·68 in 2024 (absolute increase 0·58 units); lumbar spine T-scores improved from −1·66 to −0·31 (absolute increase 1·35 units). Minor boundary effects at the most recent years reflect smoothing at the edge of the 3-year moving average window.

**Statistical Methods**

Age-standardised mean T-scores were calculated using direct standardisation. The cohort was stratified into five age bands: 40–49, 50–59, 60–69, 70–79, and ≥80 years. The pooled age distribution of the full cohort (individuals aged ≥40 years with non-missing age) was used as the reference standard. For each calendar year, age-band-specific mean T-scores were calculated and weighted by the reference age distribution to yield the age-standardised annual mean. Individuals aged under 40 years were excluded from age-standardised analyses, as low BMD in this group typically reflects secondary causes rather than secular trends in age-related skeletal health.

Three-year moving averages were applied to stabilise annual estimates for visualisation. Moving averages were calculated as unweighted means of the current, preceding, and following calendar year. Years at the boundaries of the series (1998, 1999, 2023, 2024) may show minor smoothing artefacts due to reduced averaging windows. Moving averages were used for display purposes only and did not influence model-based inference.

All analyses were conducted in R version 4·5·1. The figure was produced using the ggplot2 package within the tidyverse framework.

**Interpretation**

Both hip and lumbar spine T-scores showed sustained improvement across the study period after age standardisation, confirming that the observed decline in osteoporosis prevalence is not attributable to shifts in the age distribution of individuals referred for first DEXA scan. The larger absolute improvement at the lumbar spine (1·02 T-score units versus 0·33 at the hip) should be interpreted with caution, as lumbar spine measurements may be affected by degenerative change and vascular calcification in older adults, potentially underestimating true osteoporosis prevalence at that site.

**Methodological note**

*Age-standardised T-scores are presented as a sensitivity analysis to supplement the main logistic regression findings. They are consistent with the primary adjusted models and confirm that the observed temporal improvement is not explained by demographic shifts in the referral population.*

Supplementary Figure S1 | BMD Secular Trends Study | University Hospital Limerick & St Camillus Hospital
