# Supplementary material for: Secular trends in osteoporosis prevalence at first presentation over 26 years in a regional Irish DEXA registry: a retrospective repeated cross-sectional analysis
Source: Arch Osteoporos. 2026 Aug 19;21(1):122. doi: 10.1007/s11657-026-01764-z (PMC13490005; doi:10.1007/s11657-026-01764-z)
Supplement: Supplementary file 2 [file 11657_2026_1764_MOESM2_ESM.docx]

**Supplementary Figure S2**

**Mean age at first DEXA scan by calendar period, 1998–2024**

*Secular trends in osteoporosis prevalence at first presentation over 26 years in a regional Irish DEXA registry*

**Figure**

*Figure S2. Mean age (years) at first DEXA scan across six calendar periods (1998–2002, 2003–2007, 2008–2012, 2013–2017, 2018–2022, 2023–2024), calculated in the full first-scan cohort without age restriction (N = 66,681).* Mean age in this unrestricted cohort was approximately 60·0 years in 1998–2002, compared with approximately 61·3 years in the age-restricted (≥40 years) cohort in Supplementary Table S2 (N = 63,824); the 1·3-year difference reflects the inclusion of younger individuals in this figure. The modest and gradual increase in mean age across the study period supports the conclusion that the observed decline in osteoporosis prevalence is not primarily attributable to systematic referral of younger individuals over time.

**Purpose and Context**

A key methodological concern in registry-based secular trend analyses is that changes in referral patterns, particularly referral of younger, lower-risk individuals, could artifactually reduce observed osteoporosis prevalence over time. This figure addresses that concern directly by displaying mean age at first scan across successive calendar periods.

The modest and gradual increase in mean age across the study period (approximately 4–5 years over 26 years, with a slight plateau in the most recent period) is in the opposite direction from what would be expected if declining prevalence were driven by referral of younger individuals. If anything, the slightly older mean age in later periods would be expected to increase osteoporosis prevalence, making the observed decline more, not less, notable.

This figure complements the age- and sex-adjusted logistic regression analyses (main paper, Table 2) and the direct age standardisation (Supplementary Figure S1), providing visual confirmation that demographic shifts in the referral population do not explain the primary findings.

**Statistical Methods**

Mean age at first DEXA scan was calculated within each of six calendar periods (with the final period covering 2023–2024 only) using the full first-scan cohort without a lower age restriction (N = 66,681, excluding only individuals with missing age at scan, 0·5% of the cohort). This differs from Supplementary Table S2, which restricts to individuals aged ≥40 years (N = 63,824); the inclusion of younger individuals in this figure accounts for the slightly higher mean age observed here (approximately 61·5 years in 1998–2002) compared with 60·0 years in the age-restricted analysis. Error bars or confidence intervals are not displayed, as the large within-period sample sizes result in negligibly narrow intervals that would not be visually informative. All analyses were conducted in R version 4·5·1.

**Key finding**

*Mean age at first scan increased modestly over the study period in the unrestricted cohort (N = 66,681). This pattern is inconsistent with referral expansion toward younger, lower-risk individuals as an explanation for the observed decline in osteoporosis prevalence and supports the conclusion that the primary findings reflect genuine improvement in baseline skeletal health. The corresponding age-restricted analysis (≥40 years, N = 63,824) is shown in Supplementary Table S2 and is consistent in direction.*

Supplementary Figure S2 | BMD Secular Trends Study | University Hospital Limerick & St Camillus Hospital
