# Supplementary material for: Secular trends in osteoporosis prevalence at first presentation over 26 years in a regional Irish DEXA registry: a retrospective repeated cross-sectional analysis
Source: Arch Osteoporos. 2026 Aug 19;21(1):122. doi: 10.1007/s11657-026-01764-z (PMC13490005; doi:10.1007/s11657-026-01764-z)
Supplement: Supplementary file 3 [file 11657_2026_1764_MOESM3_ESM.docx]

**Supplementary Table S1**

**Missing data patterns stratified by age group at first DEXA scan**

*Secular trends in osteoporosis prevalence at first presentation over 26 years in a regional Irish DEXA registry*

**Table**

**Table S1. Proportion of missing values (%) for key variables, stratified by age group at first DEXA scan (overall N = 66,681).**

| **Age group** | **n** | **% missing T-hip** | **% missing T-spine** | **% missing sex** |
| --- | --- | --- | --- | --- |
| **<40** | 2,857 | 1.4 | 0.6 | 8.5 |
| **40–49** | 8,120 | 0.5 | 0.1 | 4.5 |
| **50–59** | 20,093 | 0.7 | 0.1 | 4.4 |
| **60–69** | 16,799 | 1.4 | 0.1 | 5.1 |
| **70–79** | 12,389 | 3.4 | 0.3 | 11.2 |
| **≥80** | 6,423 | **6.4** | **0.6** | **24.3** |

*Values shown as percentage of individuals within each age group with missing data for the indicated variable. Red values in the ≥80 age group indicate the highest missingness concentrations. Overall missing proportions:* hip T-score 1·9%, lumbar spine T-score 0·2%, sex 7·9%, age at scan *0%.*

**Interpretation**

Missing data were present across all variables but were modest in magnitude overall. Hip T-score missingness increased with age, from 0·5% in the 40–49 years group to 6·4% in the ≥80 years group. This pattern is clinically expected and reflects occasional omission of femoral measurements in frail older individuals where positioning may be technically difficult. It does not represent systematic data loss affecting temporal trend estimation.

Lumbar spine T-score missingness was very low across all age groups (range 0·1–0·6%), indicating near-complete ascertainment of spinal measurements throughout the study period. Sex missingness was highest in the ≥80 age group (24·3%), likely reflecting older administrative records with incomplete demographic fields. Sex values were recorded from DEXA scan.

Critically, there was no evidence of systematic differential missingness by calendar year (see main paper Methods). Logistic regression models were conducted using complete-case analysis (analytic N = 60,339 for hip and 61,267 for lumbar spine models), representing 90·5% and 91·9% of the overall cohort respectively. The complete-case analytic sample is broadly representative of the full cohort with respect to age, sex, and calendar year distribution.

**Missing data assessment summary**

*Overall missingness is low and concentrated in the oldest age group, as expected in a clinical registry of this duration. The absence of systematic temporal variation in missingness supports the validity of complete-case analysis for trend estimation. Hip T-score missingness in the* ≥80 group (6·4%) is the largest concern and is acknowledged as a limitation.

**Statistical Notes**

Missing data proportions were calculated as the number of individuals with a missing value for the indicated variable divided by the total number of individuals within each age group at first scan. The <40 years age group is included for completeness but was excluded from age-standardised analyses (Supplementary Figure S2), as BMD deficits in this group typically reflect secondary causes. All analyses were conducted in R version 4·5·1.

Supplementary Table S1 | BMD Secular Trends Study | University Hospital Limerick & St Camillus Hospital
