# Supplementary material for: Secular trends in osteoporosis prevalence at first presentation over 26 years in a regional Irish DEXA registry: a retrospective repeated cross-sectional analysis
Source: Arch Osteoporos. 2026 Aug 19;21(1):122. doi: 10.1007/s11657-026-01764-z (PMC13490005; doi:10.1007/s11657-026-01764-z)
Supplement: Supplementary file 4 [file 11657_2026_1764_MOESM4_ESM.docx]

**Supplementary Table S2**

**Baseline characteristics of individuals undergoing their first DEXA scan, stratified by calendar period**

*Secular trends in osteoporosis prevalence at first presentation over 26 years in a regional Irish DEXA registry*

**Table**

**Table S2. Baseline characteristics of individuals undergoing their first DEXA scan, stratified by 5-year calendar periods (1998–2024), restricted to age ≥40 years with non-missing age at scan (N = 63,824).**

| **Characteristic** | **Overall N** | **1998–2002** | **2003–2007** | **2008–2012** | **2013–2017** | **2018–2024†** |
| --- | --- | --- | --- | --- | --- | --- |
| **Mean age (SD)** | 63,824 | 61·3 (11·7) | 62·9 (11·8) | 63·1 (11·9) | 63·1 (11·6) | 62·7 (12·0) |
| **Female, n (%)*** | 51,685 | 94·6% | 93·9% | 88·0% | 86·1% | 84·9% |
| **Age-band distribution (n, individuals aged ≥40 years)** | | | | | | |
| *40–49 years* |  | 891 | 1,407 | 1,406 | 1,608 | 2,808 |
| *50–59 years* |  | 1,976 | 3,413 | 3,430 | 4,037 | 7,237 |
| *60–69 years* |  | 1,321 | 2,740 | 3,182 | 4,006 | 5,550 |
| *70–79 years* |  | 1,028 | 2,326 | 2,206 | 2,661 | 4,168 |
| *≥80 years* |  | 470 | 1,080 | 1,260 | 1,331 | 2,282 |
| **Period total** | **63,824** | **5,686** | **10,966** | **11,484** | **13,643** | **22,045** |

** Female percentage calculated among individuals with non-missing sex within each period. Overall female percentage (81·0%) is of the total restricted cohort (N = 63,824) regardless of sex missingness.*

*† The 2018–2024 period spans 7 years compared with 5 years for all other periods, accounting for the larger absolute counts in this column.*

*SD, standard deviation. Age-band Ns sum to period totals. Grand total across all periods = 63,824.*

**Purpose and Interpretation**

This table characterises the demographic stability of the first-scan population across the 26-year study period, focusing on individuals aged ≥40 years with non-missing age at scan (N = 63,824). It serves as the primary evidence that the observed decline in osteoporosis prevalence was not driven by systematic changes in the age or sex composition of individuals referred for DEXA assessment.

Mean age at first scan was remarkably stable across periods, ranging from 61·3 (SD 11·7) years in 1998–2002 to 63·1 (SD 11·6) years in 2013–2017, with the final period easing to 62·7 (SD 12·0) years. This near-constant mean age across more than two decades of follow-up argues against referral expansion toward younger individuals as an explanation for the declining osteoporosis prevalence. If younger, lower-risk individuals were being increasingly referred over time, a systematic decline in mean age would be expected, which is not observed.

The proportion of female individuals declined gradually from 94·6% in 1998–2002 to 84·9% in 2018–2024, reflecting a modest increase in male referrals over time, likely driven by growing clinical awareness of osteoporosis in men and expanding FRAX-based risk assessment. As males have lower osteoporosis prevalence than females, this shift in sex composition would be expected to contribute modestly to the observed decline in overall prevalence. However, the logistic regression models in the main paper adjust explicitly for sex, and the age-standardised analyses confirm that the trend persists after this adjustment.

**Key finding from this table**

*Mean age at first scan was stable across all five calendar periods* (range 61·3–63·1 years). The gradual decline in female proportion (94·6% to 84·9%) is modest and is accounted for in adjusted models. Neither demographic shift is of sufficient magnitude to explain the observed two-thirds decline in osteoporosis prevalence at first presentation.

**Statistical Notes**

This table is restricted to individuals aged ≥40 years with non-missing age at scan (N = 63,824 of 66,681 total), consistent with the age restriction applied in the direct age-standardisation analyses (Supplementary Figure S1). The <40 years group (N = 2,857) is excluded because low BMD in this age group typically reflects secondary causes rather than secular trends in age-related skeletal health.

Period totals reflect first-ever DEXA scans within each calendar period and sum to 63,824 across all five periods. The 2018–2024 period spans seven years compared with five years for all preceding periods; the larger absolute counts in this column reflect both this extended period and growing scan volume over time.

Female percentage within each period is calculated among individuals with non-missing sex. The overall female percentage (81·0%) is calculated across the full restricted cohort of 63,824 individuals irrespective of sex missingness, consistent with the figure reported in the main paper Discussion.

Supplementary Table S2 | BMD Secular Trends Study | University Hospital Limerick & St Camillus Hospital
