# Supplementary material for: Secular trends in osteoporosis prevalence at first presentation over 26 years in a regional Irish DEXA registry: a retrospective repeated cross-sectional analysis
Source: Arch Osteoporos. 2026 Aug 19;21(1):122. doi: 10.1007/s11657-026-01764-z (PMC13490005; doi:10.1007/s11657-026-01764-z)
Supplement: Supplementary file 5 [file 11657_2026_1764_MOESM5_ESM.docx]

**Supplementary Appendix S1**

**Illustrative Scenario Modelling of Potential Fracture Impact**

*Secular trends in osteoporosis prevalence at first presentation over 26 years in a regional Irish DEXA registry*

**Purpose**

This appendix provides contextual, exploratory modelling to illustrate the potential public health relevance of the observed decline in osteoporosis prevalence at first clinical presentation. The modelling translates the study's primary finding: a 65·4% relative reduction in hip osteoporosis prevalence over 26 years, into illustrative projections of future hip fracture burden under a range of assumptions.

These projections are strictly non-causal. They do not establish that the observed BMD improvements will reduce fractures; this relationship has not been empirically confirmed within the present dataset. Formal linkage to fracture and hospitalisation outcomes is ongoing.

Important: Non-causal, exploratory modelling

The projections in this appendix assume that reductions in osteoporosis prevalence at first clinical presentation may partially translate to reduced fracture risk. This assumption is plausible but unconfirmed. Results should be interpreted as illustrating the potential order of magnitude of impact, not as causal estimates.

**Methods and Assumptions**

The modelling proceeded in two steps. First, a demographic baseline trajectory was constructed projecting Irish hip fractures from a starting point of 4,000 per year (Irish Hip Fracture Database) at an assumed 2% annual increase reflecting population ageing and growth. Second, a BMD improvement factor was applied, derived from the study's adjusted odds ratio for hip osteoporosis of 0·960 per calendar year. Over 26 years (1998–2024), this corresponds to a cumulative OR of 0·346, implying an approximate 65·4% relative reduction in osteoporosis prevalence at first presentation.

The key uncertainty is the translation fraction: what proportion of this osteoporosis prevalence reduction translates to actual fracture risk reduction? Three scenarios were examined: 25% (conservative), 50% (moderate), and 75% (optimistic), spanning a clinically plausible range. No single estimate is presented as the primary finding. All projections apply the accumulated improvement as a sustained forward effect from 2024 to 2040.

**Table SA1. Modelling assumptions**

| **Parameter** | **Value / Source** |
| --- | --- |
| **Baseline hip fractures** | 4,000 per year (Irish Hip Fracture Database, IHFD) |
| **Demographic ageing trajectory** | +2% annual increase (population growth and ageing) |
| **Observed OR per year** | 0·960 (hip osteoporosis, adjusted model; main paper Table 2) |
| **Years of accumulated improvement** | 26 years (1998–2024) |
| **Cumulative OR (26 years)** | 0·346 (implying ~65·4% relative reduction in osteoporosis prevalence) |
| **Translation fractions tested** | 25% (conservative), 50% (moderate), 75% (optimistic) |
| **Projection horizon** | 2024–2040 |
| **Causal assumption** | None. Projections are exploratory only. |

**Results**

Under the demographic-only trajectory, Irish hip fractures would be expected to rise from approximately 4,000 in 2024 to approximately 5,491 by 2040. Under the three BMD improvement scenarios, projected fractures in 2040 range from approximately 4,593 (conservative, 25% translation) to 2,798 (optimistic, 75% translation), compared with the demographic-only projection of 5,491.

**Table SA2. Projected annual hip fractures by scenario, selected years**

| **Year** | **Demographic trajectory only** | **Conservative (25%)** | **Moderate (50%)** | **Optimistic (75%)** | **Fractures averted (moderate scenario)** |
| --- | --- | --- | --- | --- | --- |
| **2024** | 4,000 | 3,346 | 2,692 | 2,038 | 1,308 |
| **2028** | 4,330 | 3,622 | 2,914 | 2,206 | 1,416 |
| **2032** | 4,687 | 3,920 | 3,154 | 2,388 | 1,533 |
| **2036** | 5,073 | 4,244 | 3,414 | 2,585 | 1,659 |
| **2040** | 5,491 | 4,593 | 3,695 | 2,798 | 1,796 |

*All values rounded to nearest integer. Demographic trajectory assumes 2% annual increase from baseline of 4,000 hip fractures/year. Translation fraction: assumed proportion of observed osteoporosis prevalence reduction translating to fracture risk reduction.*

**Table SA3. Cumulative hip fractures potentially averted, 2025–2040**

| **Scenario** | **Cumulative fractures potentially averted, 2025–2040** |
| --- | --- |
| **Conservative (25%)** | 12,434 |
| **Moderate (50%)** | 24,868 |
| **Optimistic (75%)** | 37,302 |

*Cumulative fractures averted relative to demographic-only trajectory. These are potential, not predicted, values.*

**Figure**

**Figure SA1. Projected hip fractures and fractures potentially averted, 2024–2040**

*Panel A (left): Projected annual hip fractures under demographic-only trajectory (dashed) and three BMD improvement scenarios. Panel B (right): Annual fractures potentially averted relative to the demographic trajectory. Translation fraction represents the assumed proportion of the observed osteoporosis prevalence reduction translating to fracture risk reduction. These projections do not represent causal inference.*

**Limitations of this Modelling**

Several limitations should be noted when interpreting these projections.

First, the translation fraction: the proportion of the BMD improvement that reduces fracture risk is assumed rather than empirically derived. The true value is unknown and may fall outside the range tested. Second, the modelling applies the accumulated 26-year improvement as a sustained constant forward effect, which may not reflect future trajectories if referral patterns, treatment uptake, or BMD trends change. Third, fracture risk depends on multiple factors beyond BMD, including falls, frailty, and co-morbidity, none of which are captured in this model. Fourth, the baseline fracture figure of 4,000 per year is drawn from the Irish Hip Fracture Database and may not precisely represent the Midwest catchment served by this registry. Fifth, the 2% annual demographic increase is a simplifying assumption; actual demographic trends may diverge, particularly under scenarios of enhanced prevention or changing life expectancy.

These projections are intended solely to contextualise the potential order of magnitude of the study's findings for health-system planning purposes. They should not be used as the basis for specific resource allocation decisions without formal health economic modelling incorporating fracture outcomes data.

Statistical analysis note

All projections were generated in R version 4·5·1 using the tidyverse and scales packages. The full annotated R script, including all parameter definitions and output code, is available as a supplementary file accompanying this appendix.

Supplementary Table S3 | BMD Secular Trends Study | University Hospital Limerick & St Camillus Hospital
